# Gingival bleeding associated with COVID‐19 infection

**DOI:** 10.1002/ccr3.3519

**Published:** 2020-11-16

**Authors:** Rahaf Manzalawi, Khulud Alhmamey, Mohamed Abdelrasoul

**Affiliations:** ^1^ Dental Intern Dentistry Program Batterjee Medical College Jeddah Saudi Arabia; ^2^ Periodontology division Dentistry Program Batterjee Medical College Jeddah Saudi Arabia

**Keywords:** COVID‐19, dental, gingival bleeding, infectious diseases

## Abstract

Gingival bleeding, which was not previously present, may be a preceding symptom associated with COVID‐19 infections, preceding or coincidental with fever, other clinical signs, and positive testing.

## Graphical abstract

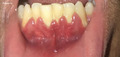

## 1 INTRODUCTION

An extensive amount of emerging data links oral manifestations as introductory signs of COVID‐19. Carreras‐Presas et al^1^ reported vesiculobullous lesions associated with the oral cavities of SARS‐CoV‐2‐infected patients. Chaux‐Bodard et al^2^ demonstrated an ulcer on the dorsal side of the tongue followed by cutaneous lesions in a 45‐year‐old female patient.

The oral cavity has been perceived as a potential reservoir for COVID‐19 asymptomatic infection, specifically the salivary glands and the oral mucosa, mostly attributed to the high expression of angiotensin‐converting enzyme‐2 (ACE2) receptors in minor salivary glands.^3^

Furthermore, clinical cases have shown signs of hyposalivation and consequent dry mouth as well as smell and taste dysfunction in several reported cases with COVID‐19 infection^4^ and.^5^

Saudi Arabia was hit hard by the COVID‐19, with cases exceeding the 150 000 cases barrier in mid‐June 2020. Nevertheless, the percentage of severe cases and mortality rates were among the lowest worldwide.^6^

A nationwide lockdown has been imposed since March and a great deal of dental clinics restricted patient reception to emergency cases after following a strict triage process beforehand.^7^

However, a number of patients depended on telephone consultations as a means of medical advice, and in a group of anecdotal cases, we noticed an association between gingival bleeding and inflammation with COVID‐19 infections.

Here, we present three patients from three different Saudi cities who reported extensive gingival bleeding and pain preceding or coincidental with the confirmation of their COVID‐19 infection. The patients underwent hospital quarantine in April and May 2020. Hence, we were not able to examine them; however, telephone communication and consultation were established with each patient, and they have contacted us with images of their gingival conditions during their quarantine time.

## 2 CASE REPORTS

### 2.1 PATIENT #1

In Taif city, a 30‐year‐old healthy male patient without any medical issues suffered from flu‐like symptoms, specifically body fatigue 2 days after coming in contact with a nurse who was diagnosed with COVID‐19 later on. Suspecting infection, he immediately reported to the hospital to undergo COVID‐19 testing; however, the hospital dismissed him because there were no signs or symptoms of COVID‐19. Nevertheless, he decided to self‐isolate.

Within 5 days of the isolation period, his temperature increased to a level that he could not cope with, and he then called the Ministry of Health (MOH) emergency phone number and reported his case. An MOH home visit practitioner took a swab. The result was positive, and he was transferred, admitted, and isolated in the hospital the same day.

Three days after testing positive, he started complaining of gingival bleeding, which deteriorated over time and was accompanied by pain, he rinsed with saline. The bleeding used to specifically increase when the patient rinsed his mouth in the early morning after bedtime on a daily basis for a period of 2 weeks during hospital quarantine.

He was treated with the standard MOH protocol with the following medications: hydroxychloroquine, ceftriaxone, and enoxaparin. The recovery started 20 days after testing positive, and signs of fatigue, fever, and gingival bleeding started to decline.

**FIGURE 1:**
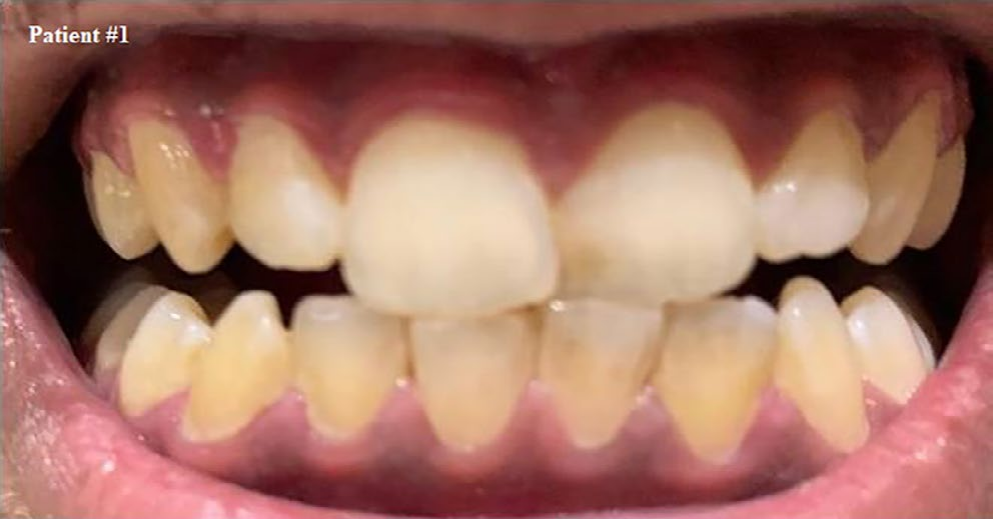
Self shot photograph of patient #1 30 d after positively testing for COVID‐19, showing residual signs of inflamed gingiva after full recovery, it slightly bleeds upon provocation but pain has completely subsided

After 30 days of testing positive, the patient showed full recovery and was discharged from the hospital. However, a slight amount of gingival bleeding was still evident, but the pain had completely resided. Figure 1

We asked the patient about his oral hygiene to investigate the reason of bleeding; he reported that he usually takes care of his teeth using a toothbrush and flosses once a day.

### 2.2 PATIENT #2

In Riyadh city, a 25‐year‐old healthy male patient without any medical issues suddenly started complaining of severe gingival pain and heavy bleeding. The pain started before any COVID‐19 symptoms appeared. Three days later, the patient suffered from fever and headache, which spanned over 4 days.

**FIGURE 2:**
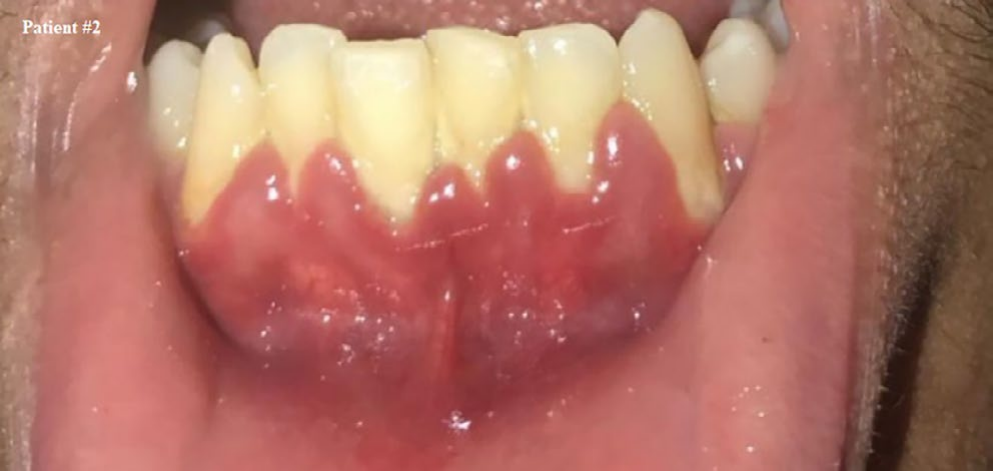
Self shot photograph of patient #2 7 d after positively testing for COVID‐19, showing inflamed gingiva with marginal redness and bulbous interdental papillae during the infection stage and quarantine period

**FIGURE 3:**
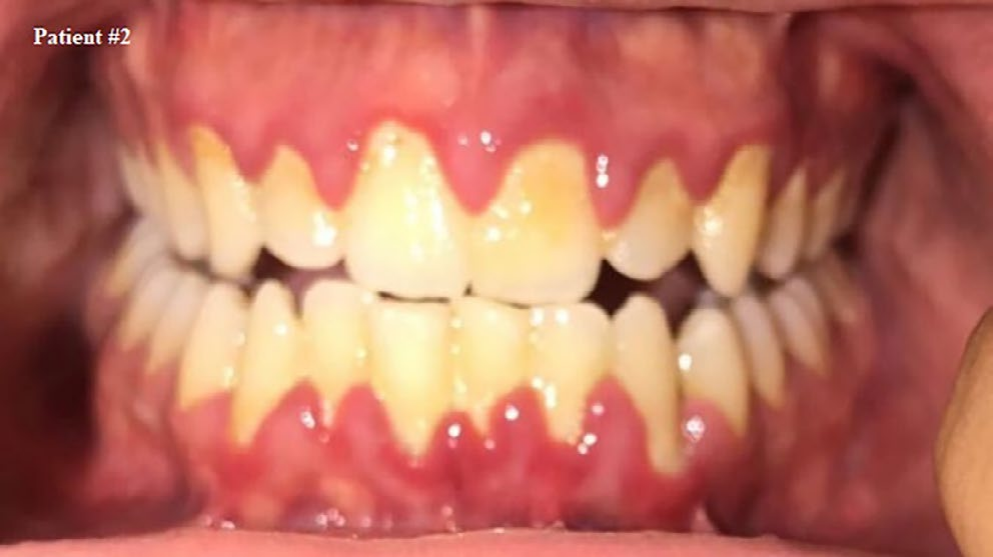
Self shot photograph of patient #2 10 d after positively testing for COVID‐19, showing persistence of gingival inflammation

With persistence of fever, he visited the hospital to undergo COVID‐19 testing and the result was positive. The patient was not aware of the source of infection and could not recall contacting symptomatic individuals. He was directed to isolate at home and was treated with paracetamol and a mouthwash (chlorhexidine). Gingival bleeding increased in a seated position. Figure 2 and Figure 3

Ten days after the confirmed positive swab, fever, and bleeding reduced. Patient started making a full recovery 3 weeks after testing positive, with small amount of gingival bleeding and much less pain. At this point, he had another swab tested and the result was negative.

The patient was asked about his oral care, and he reported that he uses a toothbrush twice a day but does not floss.

### 2.3 PATIENT #3

In Jeddah city, a 44‐year‐old healthy male banker without any medical condition received some documents by hand at his office from a client. Two days later, he started suffering from fever and gingival bleeding without any pain. He then received a call from the MOH informing him that through back tracing and tracking, they discovered that he had come in contact with a positive COVID‐19‐infected patient and was requested to test for COVID‐19. The following day, the result was positive.

**FIGURE 4:**
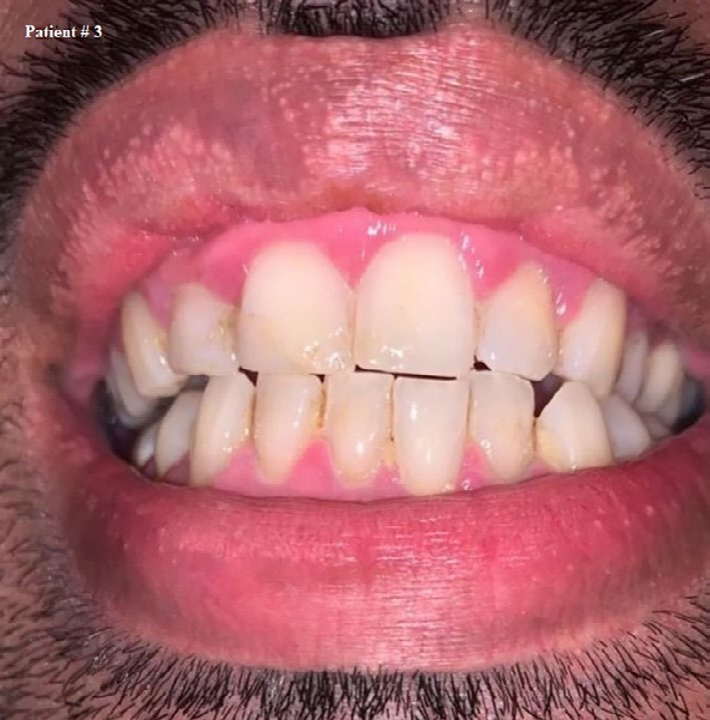
Self shot photograph of patient #3 3 d after positively testing for COVID‐19, showing shiny, bright red gingival margins

After diagnosis, the patient was isolated in a hotel facility and treated with vitamin B and natural supplements. Three days after the isolation period, fever and bleeding began to decline. Figure 4

**FIGURE 5:**
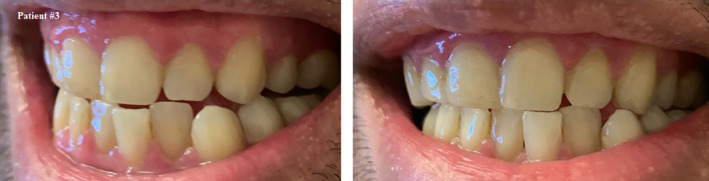
Self shot photograph of patient #3 10 d after positively testing for COVID‐19, upon recovery of symptoms showing subsiding of gingival redness

Ten days after testing positive, all the symptoms disappeared, including gingival bleeding. Figure 5 On the 12th day of isolation, another swab was taken and the result was negative,he was dismissed after 14 days since being diagnosed and isolated.

Regarding his oral care practice, he reported that he brushes his teeth once daily and that he did not suffer from gingival bleeding before being infected with COVID‐19.

## 3 CONCLUSION

Gingival and periodontal diseases are multifactorial, mostly attributed to the reaction to dental biofilm. Oral hygiene practices are integral for the prevention, treatment, and maintenance of this group of inflammatory conditions.

Debilitating disease commonly leads to neglection of proper oral hygiene measures; COVID‐19 is no exception. This leads to increased accumulation of dental biofilm, which is associated with a heightened inflammatory reaction and clinical signs of gingivitis and/or periodontitis.

Interestingly, the cases we reported had unprecedented profuse gingival bleeding that was not present before active signs of COVID‐19 developed, specifically preceding or alongside fever in common.

In the presented cases, gingival bleeding markedly declined after the infection subsided; a point worth further investigating and taken into consideration as dental clinics start opening up to cases with ease of lockdown measures in Saudi Arabia and other countries worldwide.

## CONFLICT OF INTEREST

Nothing to declare.

## AUTHORS CONTRIBUTIONS

RM and KA: conceived the idea for the study, interviewed the patients, and contributed to the write‐up of the manuscript. MA: reviewed the literature and drafted the manuscript. All authors approved the final version of this manuscript.

## ETHICAL APPROVAL

Appropriate consent has been obtained from all patients, prior to submission, in regards of the publication of images and data.

## Data Availability

All data presented and analyzed in this report are included in the published article.

## References

[1] Carreras‐Presas CM , Sánchez JA , López‐Sánchez AF , Jané‐Salas E , Somacarrera Pérez ML . Oral vesiculobullous lesions associated with SARS‐CoV‐2 infection. Oral Dis. 2020;1‐3. 32369674 10.1111/odi.13382 PMC7267423

[2] Chaux‐Bodard A , Deneuve S , Desoutter A . Oral manifestation of Covid‐19 as an inaugural symptom? J Oral Med Oral Surg. 2020;26(2):18.

[3] Xu J , Li Y , Gan F , Du Y , Yao Y . Salivary glands: potential reservoirs for COVID‐19 asymptomatic infection. J Dent Res. 2020;99(8):989. 32271653 10.1177/0022034520918518

[4] Pedrosa MS , Sipert CR , Nogueira FN . Salivary glands, saliva and oral findings in COVID‐19 infection. Pesqui Bras Odontopediatria Clín Integr. 2020; 20(supp1):e0104 10.1590/pboci.2020.112

[5] Xydakis MS , Dehgani‐Mobaraki P , Holbrook EH , et al. Smell and taste dysfunction in patients with COVID‐19. Lancet Infect Dis. 2020;20(9):1015‐1016. 10.1016/S1473-3099(20)30293-0 32304629 PMC7159875

[6] Saudi center for disease prevention and control (2020). https://covid19.cdc.gov.sa/daily‐updates/. Accessed June 16, 2020.

[7] Ministry of Health (2020). Dental Emergency protocol during COVID‐19 pandemic). https://www.moh.gov.sa/Ministry/MediaCenter/Publications/Documents/MOH‐Dental‐emergency‐guidline.pdf. Accessed June 16, 2020.

